# Evaluation of children nasal geometry, employing accoustic rhinometry

**DOI:** 10.1590/S1808-86942010000300014

**Published:** 2015-10-20

**Authors:** João Batista de Paiva, Belini Augusto Villalba Freire-Maia, José Rino Neto, Renata Cantisani Di Francesco, Richard Louis Voegels

**Affiliations:** 1 Full professor and head of the Orthodontics and Odontopediatrics Department, Dental School of the Sao Paulo University (USP); 2 Master's degree, doctoral student; 3 Associate professor, associate professor of the Orthodontics and Odontopediatrics Department, Dental School of the Sao Paulo University; 4 Doctorate, collaborating professor of the Otorhinolaryngology Discipline, Medical School, USP. Assistant physician in charge of the Pediatric Otorhinolaryngology Unit, Clinical Hospital, Medical School, USP; 5 Associate professor, associate professor at the Medical School, USP. Medical staff member of the Albert Einstein Israelite Hospital, coordinator of the University Hospital, USP

**Keywords:** acoustic, rhinometry, nasal cavity

## Abstract

The area above the nasal cavity plays a role in respiratory physiology.

**Aim:** To analyze, during a period of growth, a possible change in the minimum cross sectional area (MCA) and nasal volume of the anterior nasal cavity.

**Materials and Methods:** We evaluated 29 children (14 boys and 15 girls) with a mean age of 7.81 years at first examination (M1) and 11.27 years in the second examination (M2), without symptoms of nasal obstruction. The interval between examinations was 36-48 months. Children were subjected to the examination of acoustic rhinometry in which we recorded the minimum cross-sectional areas, volumes and their correlations with gender.

**Study design:** Cohort.

**Results:** The mean cross-sectional area of the nasal cavity of MCA for girls was 0.30 ± 0.09 cm2 (M1) and 0.30 ± 0.14 cm2 (M2), while for boys was 0.24 ± 0.12 cm2 (M1) and 0.32 ± 0.10 cm2 (M2). The mean values of the total volumes found for the whole sample were 2.17 ± 0.23 cm3 (MCA1-M1), 2.56 ± 0.27 cm3 (MCA1-M2), 4.24 ± 1.17 cm3 (MCA2- M2) and 4.63 ± 1.10 cm3 (MCA2-M2).

**Conclusion:** There was no significant change in the minimum cross sectional area of the anterior nasal cavity. There was no significant difference between genders for both MCA and for the volume. There was a significant increase in MCA1.

## INTRODUCTION

The nose has an essential role in the physiology of breathing, and is directly related with quality of life. Its inner structures filter, warm and humidify the air before it reaches the lungs. Changes in these mechanisms because of nasal block may change the predominant breathing pattern into oral rather than nasal. Other etiological factors may cause nasal problems, such as poor sleep quality or unbalanced craniofacial growth, which may also change the physiology of breathing.

Several evaluation methods to measure breathing function have been proposed. A mirrored surface placed below the nostrils measures the diameter of the halo produced by expired air; anterior rhinoscopy and the use of optic fibers, preceded by topical vasoconstrictors, has made it possible to subjectively assess the geometry of the nasal cavity (its results are examiner-dependent).^1^

Exams such as cavum radiography, teleradiography in norma lateralis, computed tomography and magnetic resonance imaging have been used in an attempt to assess breathing patency. Spiess proposed rhinomanometry in 1900; it was subsequently modified and is still used, although its subjective nature and variable results have proven unreliable.

Hilberg et al. introduced the acoustic rhinomanometer in 1989,^2^ which made the study of nasal geometry more objective and yielded reliable results. Acoustic rhinometry is a quick and easily performed non-invasive method that requires minimal cooperation from patients and yields accurate information about nasal cross-sectional areas and anterior nasal volume.^3^ Acoustic rhinometry is efficient and reliable when measuring and recognizing cross-sectional areas compared with computed tomography, which is a validated method. Evidence of this statement may be found in Mamikoglu et al.'s (2000)^4^ work, in which two methods to diagnose nasal septum deviation in the anterior turbinates were applied for intra- and inter-subject analysis. Both methods correlated less efficiently in posterior segments, although the clinical value of rhinometry remained partially in this segment.^5^ Because of the cost of computed tomography,^6^ acoustic rhinometry may be safely and reliably used in clinical practice to assess the anterior portion of the nasal cavity.

The association between predominantly vertical facial growth and mostly mouth breathing, which was made in past decades based on subjective and unreliable diagnostic exams, has added little to clarify the true effect of breathing quality on the facial pattern. The paucity of studies and objective data on nasal geometry during growth has made it hard to consistently correlate the type of facial development and breathing physiology.

The purpose of this study was to assess the minimal cross-sectional area and the volume of the nasal cavity in developing male and female healthy children.

## MATERIAL AND METHOD

The nasal cavity geometry of 29 Brazilian healthy white children (15 female and 14 male); these children were part of Paiva's (2006)^7^ sample and answered our invitation to participate. An otorhinolaryngologist evaluated all children. Inclusion criteria consisted of not having undergone any orthodontic/orthopedic treatment or otorhinolaryngological surgery for the removal of pharyngeal or palatine tonsils or inner nasal cavity structures, and nasal patency as demonstrated in rhinomanometry. Subjects with a history of abnormal nasal cavities, trauma, routine use of nasal vasoconstrictors, or recurring airway infections were excluded. At the examination patients showed no evident clinical signs of nasal inflammation. Recordings were taken at M1 (from 6.83 to 8.66 years) and M2 (from 9.83 to 12.41 years) – 36/48 months after the initial investigation. The same equipment and protocols were applied in both exams.

The institutional review board of the Dentistry School of the Sao Paulo University accepted the addendum for repeating the exams in the study sample. Opinion no. 21/01, protocol 20/01 (25/02/2008).

### Examination protocol

A RhinoScan device (RhinoMetrics A/S) was used for the rhinometric exam after each subject had acclimatized to the exam room. The exam software provides minimal cross-sectional areas and volumes in two separate points: the MCA1, which is the minimal cross-sectional area from 0 mm to 22 mm of the nostril, and the MCA2, which is located from 22 mm to 54 mm of the nostril.

Patients were comfortably seated in the appropriate position; the head was supported to avoid flexion or extension movements that would affect the quality of the exam. A previously selected adaptor was carefully placed over the right nostril without changing its shape to avoid loss of sound waves. When the exam was started, the patient was asked to hold his or her breath for sound waves to enter the nasal cavity and yield the charts in green (charts in red and yellow were not taken into account). The same sequence was applied to the left nostril.

Cross-sectional areas and volumes were obtained by adding the mean of three measurements (in green) of the minimal cross-sectional area (MCA1 and MCA2) of each nasal cavity. The total minimal cross-sectional area resulted from adding right and left values (TA); similarly for the total volume (TV).

### Statistical analysis

Two-way analysis of variance with repeated me-asures^8^ was applied where sex was the fixed factor and time was the repetition factor. A non-structured matrix was assumed for each analysis. Analyses were done separately according to the side of each nostril. Tukey's multiple comparison test^9^ was applied if the interaction term between sex and time was statistically significant; the aim was to learn whether changes occurred between one of the sexes or times.

Data were presented on mean profile charts;8 the significance level was 5%.

## RESULTS

Table 1 shows the lowest values at each time, regardless of the side. The lowest cross-sectional area was similar in M1 and M2 for males and females and across the sample (p > 0.05).

**Table 1:** Lowerst minimal cross-sectional area regardless of the side, at Moment 1 and Moment 2.

Measurement	Moment	Female	Male	Sample
		Mean SD p	Mean SD p	Mean SD p
MCA	M1	0,27 0,08	0,21 0,11	0,24 0,10
	m2	0,25 0,11 0,736	0,27 0,10 0,103	0,26 0,10 0,371

- Mean in cm^2^

- MCA – minimal cross-sectional area

Table 2 shows the mean ages and M1 and M2 measurements in different segments of the nasal cavity (MCA1 and MCA2) to the right (LD) and left (LE) and the total area for males, females and across the sample. The mean age at Moment 1 was 7.81 ± 0.62 years; the mean age at Moment 2 was 11.27 ± 0.67 years.

**Table 2:** Age and minimal cross-sectional area (cm2) at Moments 1 and 2.

Measurement	Moment		Female			Male			Sample	
		Mean	SD	N	Mean	SD	N	Mean	SD	N
MCA1 LE	M 1	0,31	0,07	15	0,33	0,10	14	0,32	0,09	29
	M 2	0,30	0,14	15	0,34	0,12	14	0,32	0,13	29
MCA1 LD	M 1	0,30	0,09	15	0,24	0,12	14	0,27	0,11	29
	M 2	0,33	0,10	15	0,32	0,10	14	0,33	0,09	29
MCA2 LE	M 1	0,41	0,11	15	0,43	0,16	14	0,42	0,13	29
	M 2	0,36	0,19	15	0,44	0,20	14	0,40	0,20	29
MCA2 LD	M 1	0,35	0,14	15	0,30	0,15	14	0,33	0,15	29
	M 2	0,42	0,13	15	0,36	0,14	14	0,39	0,13	29
MCA1 TA	M 1	0,61	0,14	15	0,57	0,16	14	0,59	0,14	29
	M 2	0,62	0,19	15	0,66	0,15	14	0,64	0,17	29
MCA2 TA	M 1	0,76	0,18	15	0,73	0,22	14	0,75	0,20	29
	M 2	0,78	0,25	15	0,81	0,24	14	0,79	0,24	29
Age (years)	M 1	7,92	0,68	15	7,69	0,56	14	7,81	0,62	29
	M 2	11,32	0,68	15	11,21	0,68	14	11,27	0,67	29

- MCA – minimal cross-sectional area

- MCA1 – 0-22mm

- MCA2 – 22-54mm

- TA – total area (LD + LE)

- M1 and M2 – Moments

**Chart 1:**
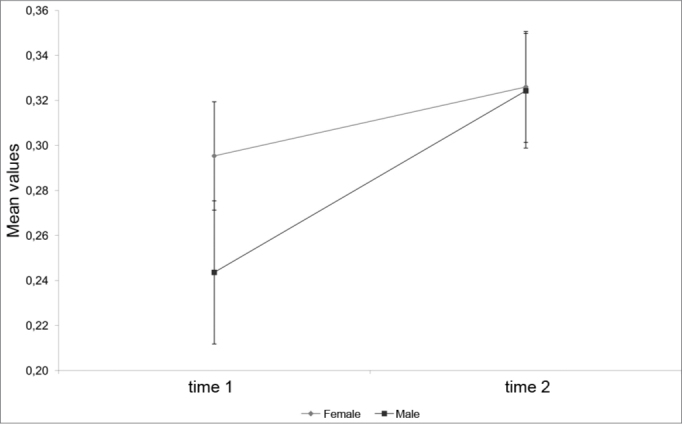
. Mean profile and standard error of the MCA1 to the right according to sex.

**Chart 2:**
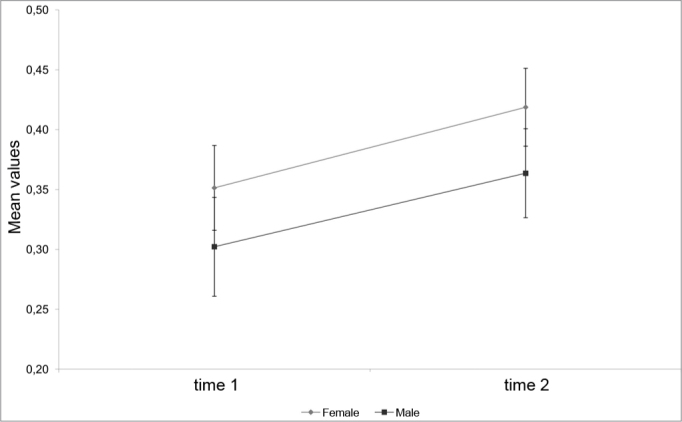
. Mean profile and standard error of the MCA2 to the right according to sex.

Table 3 shows that left side MCA1 and MCA2 measures and the total MCA1 and MCA2 area did not change between sex or time (p > 0.05). Charts 1 and 2 show that right side MCA1 and MCA2 measurements increased with time, regardless of sex (p < 0.05).

**Table 3:** Analysis of variance with repeated measures for each measurement

Measurement	Factor	gl num.	gl den.	F	p
	sex	1	27	0,84	0,369
MCA1 LE	moment	1	27	0,03	0,863
	sex* moment	1	27	0,37	0,548
	sex	1	27	0,97	0,334
MCA1 LD	moment	1	27	5,43	0,028
	sex* moment	1	27	1,10	0,305
	sex	1	27	1,08	0,309
MCA2 LE	moment	1	27	0,29	0,594
	sex* moment	1	27	0,83	0,369
	sex	1	27	1,60	0,216
MCA2 LD	moment	1	27	5,19	0,031
	sex* moment	1	27	0,01	0,918
	sex	1	27	0,00	0,955
MCA1 TA	moment	1	27	2,18	0,151
	sex* moment	1	27	1,24	0,274
	sex	1	27	0,00	0,996
MCA2 TA	moment	1	27	1,54	0,226
	sex* moment	1	27	0,50	0,484

Table 4 shows the mean nasal volumes at Moments M1 and M2 in different nasal segments (MCA1 and MCA2) to the right (LD) and left (LE) and the total area for males, females and across the sample.

**Table 4:** Description of mean nasal volumes (cm3) at M1 and M2.

Measurement	Moment		Female			Male			Sample	
		Mean	SD	N	Mean	SD	N	Mean	SD	N
MCA1 LE	M1	1,11	0,16	15	1,06	0,12	14	1,08	0,14	29
	M2	1,20	0,26	15	1,29	0,13	14	1,24	0,21	29
MCA1 LD	M1	1,11	0,14	15	1,08	0,10	14	1,09	0,12	29
	M2	1,35	0,11	15	1,30	0,15	14	1,32	0,13	29
MCA2 LE	M1	2,21	0,59	15	2,24	0,78	14	2,22	0,68	29
	M2	1,91	0,94	15	2,59	1,05	14	2,24	1,04	29
MCA2 LD	M1	2,02	0,82	15	2,01	0,94	14	2,01	0,86	29
	M2	2,63	0,62	15	2,13	0,61	14	2,39	0,65	29
MCA1 TV	M1	2,21	0,27	15	2,13	0,18	14	2,17	0,23	29
	M2	2,54	0,29	15	2,59	0,24	14	2,56	0,27	29
MCA2 TV	M1	4,23	1,11	15	4,24	1,27	14	4,24	1,17	29
	M2	4,55	1,11	15	4,72	1,13	14	4,63	1,10	29

- MCA – minimal cross-sectional area

- MCA1 – 0-22mm

- MCA2 – 22-54mm

- M1 and M2 – Moments

- TV – total volume (LD or right side + LE or left side)

Table 5 shows that left and right side MCA1 volumes on average increased statistically between M1 (p = 0.002) and M2 (p < 0.001); the same applied to total volume at MCA1 (p < 0.001).

**Table 5:** Results of analysis of variance with repeated measures for each volume

Measurement	Factor	gl num.	gl den.	F value	p
	sex	1	27	0,17	0,682
MCA1 LE	moment	1	27	11,98	0,002
	sex*moment	1	27	2,16	0,153
	sex	1	27	1,11	0,302
MCA1 LD	moment	1	27	60,90	<0,001
	sex*moment	1	27	0,06	0,809
	sex	1	27	2,02	0,166
MCA2 LE	moment	1	27	0,02	0,882
	sex* moment	1	27	2,63	0,117
	sex	1	27	1,40	0,247
MCA2 LD	moment	1	27	4,17	0,051
	sex* moment	1	27	1,80	0,191
	sex	1	27	0,07	0,794
MCA1 TV	moment	1	27	44,28	<0,001
	sex*moment	1	27	1,07	0,310
	sex	1	27	0,07	0,790
MCA2 TV	moment	1	27	2,82	0,105
	sex*moment	1	27	0,12	0,729

There were no statistically significant time differences in MCA2 (p > 0.05), and no differences in volumes between sexes (p > 0,05); the behavior of volumes according to sex across the times was statistically equal (p interaction > 0.05).

## DISCUSSION

It has been suggested that an altered breathing pattern, from nasal to oral, is one of the factors causing unbalanced facial growth.^10^, ^11^, ^12^ Studies such as those by Harvold in 1973^13^ have given credibility to this statement. Quantifying the influence of breathing on facial growth remains an object of research, since there is no consensus in the literature about the methods used in such studies.

Airflow through the nasal cavity into the lungs may face obstructions along its path. Handelman^13^ stated that the size of the nasopharynx increases by 80% to 150% during growth because the nasopharyngeal area increases in size and the lymphoid tissue of the pharyngeal tonsils decreases; nasopharyngeal growth ceases in girls at the age 13 to 14 years and in boys at about age 18 years. But what happens in the anterior nasal cavity? This study aimed to assess the minimal cross-sectional area and volume of the anterior nasal cavity (from 0,0 mm to 54 mm of the nostril) by using MCA1 and MCA2 areas, in a 36 to 48 month interval (M2), during growth and development; it also aimed to check for any sex difference. Data on 100 children in a study by Paiva^7^ were used as initial parameters (M1). Participants of that study were invited to have the exams repeated, according to the same study protocol of the initial investigation. Our sample comprised 29 children; other were excluded because of orthodontic and/ or otorhinolaryngological treatment between M1 and M2, which would mask spontaneous growth. Other children did not participate because they were lost to contact or did not wish to participate. Notwithstanding the difficulties of a longitudinal study (3-4 years), we were able to reassess 29% of the sample in the initial study; this sample number was statistically sufficient for the present study, and is in line with other published studies.^1^,^14^, ^15^, ^16^

We assessed the minimal cross-sectional area (MCA) based on the MCA1 and MCA2 references in three conditions: the anterior nasal cavity MCA, whether right or left; the MCA according to each side (right and left); and the total MCA, which is the sum of the right and left nasal cavity areas (MCA TA). The same was done for volume.

The smallest cross-sectional area of the anterior nasal cavity in females, regardless of the side, was 0.27 ± 0.08 cm^2^ at M1 and 0.25 ± 0.11 cm^2^ at M2; it was M1 0.21 ± 0.11 cm^2^ at M1 and 0.27 ± 0.10 cm^2^ at M2 in males, as shown on Table 1. The mean MCA at both study Moments was 0.26 ± 0.10 cm2 in females, and 0.24 ± 0.11 cm^2^ in males. These numbers are close to those presented by Vig and Zajac^17^ in an American sample aged from 5 to 12 years: 0.32 cm^2^ in females and 0.30 cm^2^ in males. The −0.02cm^2^ difference in females (p = 0.736) and the 0.06 cm^2^ difference in males (p = 0.103) between M1 and M2 was not significant.

Minimal cross-sectional area values of the anterior nasal cavity in the sample, regardless of the side, was 0.24 ± 0.10 cm^2^ at M1 and 0.26 ± 0.10 cm^2^ at M2, as shown on Table 1. This change was statistically significant (p= 0.371). The mean MCA-Sample value from M1 to M2 was 0.25 ± 0.10 cm.^2^ Carlini^1^ studied children aged 7 to 13 years and found an MCA of 0.35 cm.^2^ Comparing this value with the mean MCA in our study yields a 0.10 cm^2^ difference. Carlini's^1^ higher value may be associated with the age range of her sample (children up to age 13 years).

Analysis of the total minimal cross-sectional area -MCA-TA (right and left nasal cavities) – in females revealed 0.61 cm^2^ at M1 and 0.62 cm^2^ at M2. In males, the MCA-TA was 0.57 cm^2^ at M1 and 0.66 cm^2^ at M2. There was, therefore, a 0.01 increase in females and a 0.09 increase in males, which is shown on Table 2.

The minimal cross-sectional area of the anterior nasal cavity in the sample (MCA-TA sample) was 0.59 cm^2^ at M1 and 0.64 cm^2^ at M2. There was a 0.05 cm^2^ increase. The mean MCA-TA value between M1 and M2 was 0.62 cm.^2^

The right and left nasal cavity MCA, measured separately, yielded different MCA values in the anterior nasal cavity (Table 1), because a lower MCA may be in the right or left nasal cavity, as seen when comparing Table 1 with Table 2.

Table 2 shows the right and left nasal cavity MCA, where its value is 0.27 cm^2^ at M1 and 0.32 cm^2^ at M2. This 0.05 cm2 increase was not significant, as seen on Table 3. Analyzing only the right or left nasal cavity MCA1 or MCA2 at both study Moments (M1 or M2) reveals a significant gender-independent increase, as seen in the right MCA1 and MCA2, on Table 2 (p<0.05). Analyzing the left MCA2 at Moments M1 and M2 on Table 2 reveals a decreased albeit not significant area, as seen on Table 3 (p > 0.05).

Crouse et al.18 has reported a decreased MCA in a study of children aged from 9 to 13 years; these authors found slightly higher MCA values at age 9 years compared to 10 years. They added that the lowest MCA values occurred at age 10 years in both males and females, attributing this finding to an altered nasal mucosa.

We found a mean 0.02 cm^2^ increase in the MCA regardless of the side (Table 1), a mean 0.05 cm2 increase when analyzing the right and left nasal cavities separately and a mean 0.05cm^2^ increase in the analysis of the MCA-TA sample (Table 2). Crouse et al.18 have reported similar values at ages 9 to 12 years (0.06 cm2 increase). Their method, however, differed from our approach; these authors calculated the MCA by applying a mathematical equation using nasal flow, air density, and oral pressure difference values, gathered by the use of a pneumotachograph and mask.

Warren et al.^19^ have stated that the MCA in normal adults should be 0.60cm.^2^ Warren had first suggested this MCA value in 1969.^20^ These authors suggested that adults with an MCA below 0.40 cm^2^ (33% decrease in the minimal nasal cross-sectional area) would present an increased airway resistance and worsened nasal breathing. Their investigation of the effect of age on the MCA of children yielded 0.21 ± 0.05 cm^2^ at age 6 years, increasing to 0.46 + 0.15 cm2 at age 14 years, a 0.032 cm^2^ MCA increase each year. Laine & Warren^21^ have suggested that adult MCA values are reached at ages 15 to 16 years. These values range from 0.050 to 0.60 cm^2^ according to Laine-Alava & Minkkinen,^22^ Warren et al.,^19^ Vig & Zajac,^17^ and Huggare & Laine-Alava.^23^

We found no statistically significant differences in MCA between males and females (Table 3). Laine & Warren,^21^ Laine-Alava & Minkkinen,^22^ Vig & Zajac,^17^ Ellingsen et al.,^24^ and de Straszek et al. have also described the same result.^25^ Corey et al. in 1998^26^ noted that this difference may be present after puberty. On the effect of age over the MCA, Laine & Warren^21^ have noted that this was more evident in later stages of their longitudinal study; they found that the MCA increased from 0.038 ± 12cm^2^ to 0.046 ± 16 cm^2^ from ages 7 to 15 years. Crouse et al.^18^ found a 0.05cm^2^ increase from ages 12 to 13 years, which was almost the same increase between ages 9 and 12 years. Zavras et al.^14^ have stated that age-based selection criteria may yield false results because the rate of growth varies individually.

Table 4 shows the total volume of the nasal valve region (MCA1 TV); this value in females was 2.21cm^3^ at M1 and 2.54 cm^3^ at M2. In males, these values were 2.13 cm^3^ at M1 and 2.59 cm^3^ at M2. There was a 0.33 cm^3^ increase in females and a 0.46 cm3 increase in males, which was statistically significant (Table 5). The value for the entire sample was 2.17 cm^3^ at M1 and 2.56 cm^3^ at M2, a 0.39 cm3 increase between moments, which was also statistically significant (Table 5). For the MCA2 the volumes for the entire sample were 4.24 cm^3^ at M1 and 4.63 cm^3^ at M2. There was a 0.39 cm^3^ increase, which was not statistically significant (Table 5). Our values for this region (22-54 mm) are lower than those found by Millqvist and Bende^27^ in children of similar age. Those authors found 5.66 cm^3^ in the first evaluation and 6.54 cm^3^ two years later, a 0.88 cm^3^ increase.

Different from the minimal cross-sectional area at MCA1 and MCA2, where we found a significant increase between M1 and M2 only to the right, the volumes for both sides and the total volume were statistically significantly increased at MCA1 between both moments (Table 5).

As in the MCA, we found no gender differences in volume, which concurs with the findings of Millqvist and Bende.^27^

The anterior nasal cavity is one of the regions that define breathing patterns. It is essential to establish the minimal cross-sectional area of this region – which is a path for adequate airflow for bodily oxygenation – if we wish to understand the effect of the complex respiratory system on craniofacial growth, and thereby the quality of life of patients. In this study, the lowest area was found at MCA1, which is the first 22 mm of the nasal cavity and contains the nasal valve.

Breathing quality is closely associated with the areas through which air flows. The nasal septum divides the nasal cavity into its right and left side, which are both analyzed. Inspired air, which initially enters the nose separately, meets at the nasopharynx and continues to the lungs. A lower than normal minimal cross-sectional area on one side may be compensated by an enlarged other side to yield the same air volume for bodily oxygenation. Thus, a lower minimal cross-sectional area on one side does not necessarily change predominantly nasal breathing into mouth breathing; if so, it will not affect vertical facial growth.

## CONCLUSION

– There was no significantly increased minimal cross-sectional area at MCA1 and MCA2 to the left.
– There was a significantly increased minimal cross-sectional area at MCA1 and MCA2 to the right.
– There was not significantly increased minimal cross-sectional area when the total area was assessed.
– There was a significantly increased volume at MCA1 to the right and left, and a significantly increased total volume.
– There was no significantly increased volume at MCA2 to the right and left, and no significantly increased total volume.
– No significant gender differences were found in nasal geometry.

## References

[1] Carlini D. (1999). Rinometria acústica na avaliação de pacientes entre 7 e 13 anos de idade com obstrução nasal por rinite crônica hipertrófica não infecciosa (Dissertação de Mestrado).

[2] Hilberg O., Jackson A.C., Swift D.L., Pedersen O.F. (1989). Acoustic rhinometry evaluation of nasal cavity geometry by acoustic reflection. J Appl Physiol..

[3] Fonseca M.T., Goto E.Y., Nigro C.E.N., Rocha F.M., Mello Júnior J.F., Voegels R.L. (2003). Reprodutibilidade e repetibilidade da rinometria acústica. Arq Otorrinolaringol..

[4] Mamikoglu B., Houser S., Akbar I., Ng B., Corey J.P. (2000). Acoustic rhinometry and computed tomography scans for the diagnosis of nasal septal deviation, with clinical correlation. Otolaryngol Head Neck Surg..

[5] Terheyden H., Maune S., Mertens J., Hilberg O. (2000). Acoustic rhinometry: validation by three-dimensionally reconstructed computer tomographic scans. J Appl Physiol..

[6] Corey J.P., Gungor A., Nelson R., Fredberg J., Lai V. (1997). A comparasion of the nasal cross-sectional áreas and volumes obtained with acoustic and magnetic resonance imaging. Otolaryngol Head Neck Surg..

[7] Paiva J.B. (2006). Estudo comparativo da geometria nasal e da resistência respiratória em diferentes tipos faciais (Tese de Livre Docência).

[8] Singer J.M., Andrade D.F., Sen P.K., Rao C.R. (2000). Handbook of Statistics. Volume 18: Bio-Environmental and Public Health Statistics.

[9] Neter J, Kutner MH, Nachtsheim CJ, Wasserman W. Applied Linear Statistical Models. 4. ed. Illinois; 1996.p.1408.

[10] Ricketts R.M. (1968). Respiratory obstruction syndrome. Am J Orthod..

[11] Harvold E.P., Vargervik K., Chierici G. (1973). Primate experiments on oral sensation and dental malocclusions. Am J Orthod..

[12] Linder-Aronson S. (1979). Respiratory function in relation to facial morphology and the dentition. Br J Orthod..

[13] Handelaman C.S., Osborne G. (1976). Growth of the Nasopharynx and Adenoid Development from One to Eighteen years. Angle Orthodont..

[14] Zavras A.I., White G.E., Rich A., Jackson A.C. (1994). Acoustic rhinometry in the evaluation of children with nasal or oral respiration. J Clin Pediatr Dent..

[15] Marhioro E.M. (1999). Efeito da expansão rápida da maxila na cavidade nasal avaliado por meio da rinometria acústica (Tese de Doutorado).

[16] Trindade I.E.K., Gomes A.O.C., Sampaio-Teixeira A.C.M., Trindade S.H.K. (2007). Volumes nasais de adultos aferidos por rinometria acústica. Rev Bras Otorrinolaringol..

[17] Vig P.S., Zajac D.J. (1993). Age and gender effects on nasal respiratory function in normal subjects. Cleft Palate Craniofac J..

[18] Crouse U., Laine-Alava M.T., Warren D.W., Wood C.L. (1999). A longitudinal study of nasal airway size from age 9 to age 13. Angle Orthod..

[19] Warren D.W., Hairfield W.M., Dalston E.T. (1990). Effect of age on nasal cross-sectional area and respiratory mode in children. Laryngoscope..

[20] Warren D.W., Duany L.F., Fischer N.D. (1969). Nasal pathway resistance in normal and cleft lip and palate subjects. Cleft Palate J..

[21] Laine T., Warren D.W. (1991). Effects of age, gender, and body size on nasal cross-sectional area in children. Eur J Orthod..

[22] Laine-Alava M.T., Minkkinen U.K. (1997). Variation of nasal respiratory pattern with age during growth and development. Laryngoscope..

[23] Huggare J.A., Laine-Alava M.T. (1997). Nasorespiratory function and head posture. Am J Orthod Dentofacial Orthop..

[24] Ellingsen R., Vandevanter C., Shapiro P., Shapiro G. (1995). Temporal variation in nasal and oral breathing in children. Am J Orthod Dentofacial Orthop..

[25] Straszek S.P., Moeller A., Hall G.L., Zhang G., Stick S.M., Franklin P.J. (2008). Reference values for acoustic rhinometry in children from 4 to 13 years old. Am J Rhinol..

[26] Corey J.P., Gungor A., nelson R., Liu X., Fredberg J. (1998). Normative standards for nasal cross-sectional areas by race as measured by acoustic rhinometry. Otolaryngol Head Neck Surg..

[27] Millqvist E., Bende M. (2006). Two-year follow-up with acoustic rhinometry in children. Am J Rhinol..

